# Synthetic CT‐enabled weekly adaptive radiotherapy for nasopharyngeal carcinoma: Optimizing plan adaptation triggers through volumetric–dosimetric monitoring

**DOI:** 10.1002/acm2.70676

**Published:** 2026-07-01

**Authors:** Zhenmei Cao, Chun Wu, Lixiang Han, Tingting Duan, Fengyu Zhang, Lansheng Zhang, Bin Wang, Weijie Lei, Xinye Ni

**Affiliations:** ^1^ Department of Radiotherapy, Second People's Hospital of Changzhou Third Affiliated Hospital of Nanjing Medical University Changzhou China; ^2^ Department of Radiation Oncology Second Affiliated Hospital of Xuzhou Medical University Xuzhou China; ^3^ Department of Radiation Oncology Xuzhou First People's Hospital Xuzhou China

**Keywords:** adaptive radiotherapy, dosimetric variations, nasopharyngeal carcinoma, replanning triggers, synthetic CT

## Abstract

**Background and purpose:**

Anatomical changes during radiotherapy for nasopharyngeal carcinoma (NPC) frequently compromise target volume coverage while escalating dose delivery to organs at risk (OARs). In adaptive radiotherapy (ART), optimizing workflow efficiency to minimize clinical workload while maximizing patient benefit remains a fundamental challenge. Previous investigations inadequately tackled the critical aspects of identifying robust triggers and optimal timing for ART initiation. To bridge this gap, we quantified weekly sCT‐derived volumetric and dosimetric changes on a C‐arm linac workflow to determine robust, literature‐based thresholds and optimal replanning time points for NPC ART.

**Materials and methods:**

This study analyzed 52 NPC patients undergoing VMAT. Weekly cone‐beam CT (CBCT) scans (*n* = 312) were processed through ArcherQA using a CycleGAN‐based sCT generation pipeline, which enabled automated segmentation and GPU‐accelerated Monte Carlo dose recalculation. Longitudinal volumetric/dosimetric changes in targets and organs at risk (OARs) were tracked, with Spearman correlation analyzing variable relationships. Binary logistic regression and Receiver Operating Characteristic (ROC) curve analysis identified evidence‐based triggers and optimal timing for adaptive replanning, enabling quantitative criteria for ART initiation while balancing precision and workflow efficiency.

**Results:**

Anatomical changes drove dosimetric deviations: PGTVn (nodal targets) showed ≥8% volume reduction by week 2 (OR = 2.03, *p* = 0.006, AUC = 0.83), while PGTVp/PTV1 (primary targets) required ≥10% reduction by week 4 (AUC = 0.76). OAR correlations included left submandibular gland volume inversely linking to mean dose (peak *r* = ‐0.575 at week 3, *p* < 0.001) and delayed ipsilateral parotid atrophy (week 4). Triggers were defined: ≥15% contralateral parotid reduction at week 2 (AUC = 0.89) and ≥19% left submandibular gland loss at week 3 (AUC = 0.97). Pharyngeal constrictor analysis revealed that week 2 ≥4% volume loss predicted ≥0.9 Gy week 3 dose rise (AUC = 0.93).

**Conclusion:**

Weekly sCT monitoring clarified the optimal timing for offline ART, revealing that structure‐specific dosimetric changes necessitate distinct intervention windows, notably week 2 for nodal/contralateral parotid and week 4 for primary/ipsilateral parotid, providing a practical framework for prospective validation.

## 1 INTRODUCTION

Nasopharyngeal carcinoma (NPC) represents a unique clinical entity among head and neck cancers,^1^ characterized by its high radiosensitivity and intricate anatomical proximity to critical structures such as the salivary glands (SG).^1^, ^2^ While intensity‐modulated radiotherapy (IMRT) and volumetric modulated arc therapy (VMAT) have markedly improved dose conformality, patients frequently experience significant anatomical changes, including tumor shrinkage, weight loss, and organ deformations, throughout the treatment course.^3^, ^4^ These interfractional variations can lead to dosimetric deviations, compromising target coverage and increasing the risk of radiation‐induced toxicities, notably xerostomia.^5^, ^6^

Adaptive radiotherapy (ART) is posited as a pivotal strategy to mitigate these effects by modifying the treatment plan in response to anatomical changes.^7^, ^8^, ^9^ However, the conventional offline ART workflow, dependent on intermittent replanning CT (pCT) scans, is clinically resource‐intensive and often fails to capture the dynamic nature of anatomical alterations at optimal adaptive timing, risking suboptimal treatment outcomes.^10^ While emerging online or real‐time ART solutions on specialized platforms, such as a ring‐mounted cone‐beam computed tomography (CBCT)‐guided radiotherapy unit (e.g., Ethos) or advanced hybrid systems like a magnetic resonance linear accelerator (e.g., Unity) can address this latency by adapting the plan immediately prior to or during treatment, they introduce other significant challenges. These solutions often demand substantial human intervention for contour review and plan approval, increasing the clinical workload. Moreover, the prolonged patient immobilization required for these complex on‐couch workflows not only heightens patient discomfort but also increases the risk of intra‐fractional motion, potentially compromising the final delivery accuracy.^11^, ^12^ The CBCT equipped on a c‐arm linac provides a readily available solution for daily imaging, yet its inherent image artifacts, such as scatter, noise, and beam hardening, hinder its direct application for accurate contouring and dose calculation.^13^, ^14^ Consequently, there is a pressing clinical need for a streamlined, automated methodology that leverages routine CBCT imaging to enable frequent and precise assessment, thereby facilitating data‐driven decisions on the optimal timing for plan adaptation. Recent advancements in deep learning (DL) have facilitated the generation of sCT from CBCT, offering a promising avenue to overcome the limitations of CBCT.^15^, ^16^, ^17^ In our previous study, Lei et al.^18^ established the foundational feasibility of this approach for NPC ART on a conventional C‐arm linac. By employing a cycle‐consistent generative adversarial network (cycleGAN), we demonstrated that sCTs could achieve high geometric (Dice similarity coefficient, DSC > 0.85 for most structures) and dosimetric agreement with pCTs, while also revealing strong correlations between sCT‐derived volumetric changes and offline ART plan adjustments, particularly for the parotid glands.^18^ Nonetheless, this study lacked the continuous, high‐frequency monitoring data afforded by weekly CBCT.

Building upon this groundwork, subsequent research has sought to define the optimal timing and triggers for ART replanning. Several studies, such as those by Gan et al.^19^ and Huang et al.,^20^ have proposed replanning timepoints based on pCT scans, suggesting interventions around the third to fourth week or within the first three weeks, respectively. Others, like Aristophanous et al.,^21^ have developed offline decision‐support tools using deformable image registration (DIR) to identify candidate triggers, including a parotid volume reduction >7%, a neck volume reduction >2%, and GTVn reduction >29%. Complementing these anatomical approaches, Lim et al. ^22^ investigated an innovative indirect surrogate metric by leveraging a real‐time in vivo portal dosimetry system, demonstrating that changes in transit fluence could be associated with a pre‐defined 5% volume reduction trigger (AUC = 0.88). Notably, their study^22^ also suggested that target volume changes were correlated with variations in OARs, either volumetrically or dosimetrically. Although these studies provide valuable insights into the dynamics of ART, there remain areas for further refinement to enhance clinical applicability. The proposed triggers predominantly rely on volumetric changes alone, often derived from limited pCT acquisitions or semi‐automated DIR workflows, which may not fully capture the resultant dosimetric impact. More importantly, the common reliance on a single, static threshold (e.g., 5% or 29% volume reduction) failed to account for the dynamic and continuous nature of anatomical changes throughout treatment. In a scenario resembling oART, where target volumes may fluctuate fraction‐by‐fraction, occasionally enlarging due to edema or shrinking due to therapeutic response, a trigger mechanism based on a fixed threshold value could prove either excessively sensitive or insufficiently responsive.^23^ Leveraging the evolving correlation between target volume dynamics and OAR variations may enable more robust adaptation decisions by accounting for the continuous, fraction‐dependent interplay of anatomical changes.

To address the aforementioned gaps, this retrospective study aimed to establish evidence‐based, weekly volumetric and dosimetric triggers for adaptive replanning in NPC by leveraging a comprehensive sCT‐enabled monitoring workflow. A cohort of 52 patients with stage II–IV NPC was analyzed, from whom a total of 312 cone‐beam CT (CBCT) scans were acquired during treatment. The ArcherQA platform, integrated with a DL‐based framework, generated weekly sCTs from CBCT scans. This enabled automated contouring with manual refinement and highly precise GPU‐accelerated Monte Carlo dose recalculation. Our analysis focused on quantifying the weekly trajectories of volumetric changes for both target volumes (PGTVp, PGTVn, PTV1, PTV2) and key OARs (parotid, submandibular glands, oral cavity, pharyngeal constrictor muscles), along with their corresponding dosimetric deviations (e.g., D98, D2, V95 for targets; mean dose for OARs). By systematically analyzing these longitudinal, sCT‐derived data, this study aimed to identify literature‐informed critical thresholds and optimal timing for plan adaptation, thereby providing a practical framework for implementing ART in NPC radiotherapy on conventional C‐arm linacs.

## 2 METHODS

### 2.1 Patient characteristics

This study retrospectively analyzed 52 patients with stage II‐IV nasopharyngeal carcinoma (NPC) patients treated with VMAT on a C‐arm linear accelerator (Varian VitalBeam, USA) between December 2018 and March 2025. A total of 312 CBCT scans were acquired during treatment. All patients had pathologically diagnosed squamous cell carcinoma (SCC). The tumor stages were classified according to the AJCC 8th edition criteria. The median age was 58 years (range: 16 ‐ 84).

The cohort included 32 male and 20 female patients. Patients with consecutive missing CBCT scans for ≥2 treatment weeks were excluded. Patient characteristics are summarized in Table 1.

**TABLE 1:** Patients’ characteristics.

Clinical characteristics	No. of patients	%
Gender		
Male	32	61.54
Female	20	38.46
Age(ages)		
Range	16–84	
Median	58	
T stage		
T1	9	17.31
T2	19	36.54
T3	14	26.92
T4	10	19.23
N stage		
N0	1	1.92
N1	9	17.31
N2	28	53.85
N3	14	26.92
Total stage		
I	—	—
II	9	17.31
III	23	44.23
IV	20	38.46
Definitive radiotherapy	3	5.77
Neoadjuvant chemotherapy	20	38.46
Concurrent chemotherapy	27	51.92
Adjuvant chemotherapy	2	3.85

### 2.2 Localization and CT simulation

All patients underwent planning CT simulation in the supine position using a radiotherapy‐dedicated large‐bore CT scanner (Discovery™ RT, GE Healthcare, USA) with indexed thermoplastic immobilization, the longitudinal range from the Vertex (+2 cm margin) to the carina (T5 vertebral level), with a reconstructed slice thickness of 2.5 mm and contiguous slice spacing, which was imported into the Eclipse™ treatment planning system (TPS, version 13.6, Varian Medical System, Palo Alto, Calif) for structure delineation and VMAT Optimization.

### 2.3 Definition of TVs and planning delivery

Based on the CT and MR fusion images, targets and OARs were delineated in the Eclipse system by board‐certified radiation oncologists with >10 years of subspecialty experience in NPC radiotherapy, adhering to consensus guidelines,^24^, ^25^ with adjudication by a second expert in cases of discordance. The target volume delineated contains the primary tumors (GTVp) and the lymph nodes (GTVn), the high‐risk target volume (CTV1), and the low‐risk target volume (CTV2) areas. Building upon the above delineated target volumes, the planning target volumes (PTVs) were derived through evidence‐based margin expansions, defined as PGTVp, PGTVn, PTV1, and PTV2. Structures such as the spinal cord, brain stem, optic nerve, lens, optic chiasm, eyes, parotid glands (PG), submandibular glands (SG), larynx, oral cavity (OC), pharyngeal constrictors (PCM), mandible and temporal lobe were included.

The dose prescription for the PGTVp, PGTVn, PCTV1, and PCTV2 were 69.96 Gray (Gy) in 33 fractions (Fx, 2.12 Gy per fraction), 69.96 Gy in 33 Fx (2.12 Gy per fraction), 60.06 Gy in 33 Fx (1.82 Gy per fraction), and 50.96 Gy in 28 Fx (1.82 Gy per fraction), respectively. Dose optimization prioritizes sparing of organs at risk (OARs), especially the PGs, OC, SGs, and PCM, while maintaining adequate target coverage and adhering to dose constraints for critical neural structures, including the spinal cord and brainstem. VMAT optimization in Eclipse TPS ensured that 95% of the PTV is adequately covered by 100% of the prescribed dose.

### 2.4 CBCT scan and synthetic CT generation

All VMAT treatments were delivered using a Varian VitalBeam linear accelerator, with pretreatment CBCT verification performed at every fraction to confirm patient positioning accuracy. Weekly CBCT scans were acquired at fractions 1(week1), 7(week2), 12(week3), 17(week4), 22(week5), and 27 (week6) for image‐guided radiotherapy (IGRT) verification. The CBCT datasets, along with the reference planning CT, RT structure sets, and RT dose distributions, were systematically imported into the Archer QA system for sCT generation (sCT1, sCT2, sCT3, sCT4, sCT5, sCT6). All automatically generated target contours were carefully reviewed, verified, and manually adjusted by two senior radiation oncologists with over 10 years of subspecialty experience in NPC radiotherapy. The detailed workflow of sCT generation, contouring protocol and dose calculation accuracy had been systematically validated in our previous study.^18^

### 2.5 Missing data imputation

To maintain data quality, subjects missing CBCT scans for ≥2 consecutive treatment weeks were excluded from the study. The K‐nearest neighbors (KNN) algorithm (implemented via R package “DMwR”; k = 5, weighted average method) was employed for imputing missing weekly Dmean and volumetric measurements of targets and OARs, as it demonstrated significantly better performance than multivariate imputation by chained equations (MICE) in preserving dose distribution accuracy based on prior validation studies.^19^

### 2.6 Analysis metrics

Dose‐volume histogram (DVH) data were systematically collected for all target volumes and organs at risk (OARs). Six comparative plans (designated Plan1‐Plan6) were generated using sCT images derived from registered original CT datasets through recalculation.

Volumetric Changes: The volumetric changes of targets (PGTVp, PGTVn, PTV1, and PTV2) and OARs (PGs, OC, SGs and PCM) were quantified weekly. For the PG, we also classified as ipsilateral (PG_I) or contralateral (PG_C) based on the involvement of Level II cervical lymph nodes. If Level II lymph nodes were positive on either side, the corresponding PG was designated as ipsilateral; alternatively, if no Level II lymph node metastasis was present, both PGs were considered contralateral.^3^ Serial weekly CBCT‐derived neck structure volumetric changes may serve as a surrogate biomarker for weight loss during radiotherapy.^22^ Specific metrics included volumetric structure changes (V%) between Plan1 (reference) and Plans2‐6 were systematically evaluated.

Dosimetric Changes: The mean dose (Dmean) to the PGs, OC, SGs and PCM, the targets (PGTVp, PGTVn, PTV1, and PTV2) D98, D2, and V95 were evaluated.

### 2.7 Statistical analysis

All statistical analyses were conducted using SPSS v26.0 with two‐tailed *p*‐values < 0.05 considered statistically significant.

Friedman Test: Nonparametric tests were employed due to non‐normal data distributions (Shapiro‐Wilk test, all *p* < 0.05). The Friedman test (nonparametric repeated‐measures ANOVA) was used to identify longitudinal trends in volumetric and dosimetric parameters (Plan1 to Plan6). When significant (*p* < 0.05), post‐hoc pairwise comparisons were performed using Wilcoxon signed‐rank tests with Bonferroni correction. Median differences with Hodges‐Lehmann 95% confidence intervals were reported for all Wilcoxon test results.

Spearman's Rank Correlation: To assess whether volumetric structure changes (V%) could serve as potential triggers for ART, we performed Spearman rank‐order correlation analyses between V%​and the resulting dosimetric impact (△D) on both OARs and PTVs. The relative volume changes (V%) between Plans 2–6 relative to Plan1 (measured via ArcherQA) were calculated as: V% = (V_i−_V_1_)/V_1 _× 100%.

Binary Logistic Regression and ROC Analysis: To establish a clinically relevant reference standard for evaluating the predictive performance of sCT‐derived volumetric changes, we first defined a binary endpoint (“replanning required” vs. “not required”) based strictly on pre‐established thresholds derived from external peer‐reviewed literature.^5^, ^14^, ^15^, ^19^, ^21^, ^26^, ^27^ While these criteria were externally sourced to maintain standardization, they provide a relevant reference framework for analyzing the sCT‐derived volumetric predictors in the context of offline ART. Specifically, a patient was classified as requiring replanning if any of the following conditions were met:

1. For OARs: a volume reduction of ≥20% in the PG or SG, or a dose increase of ≥3 Gy;^19^ or a >5% volume reduction or a > 1 Gy dose increase in the pharyngeal constrictor muscles (PCM).^26^
2. For targets: a volume reduction of ≥10% in the PGTVp, PGTVn, PTV1, or PTV2;^5^, ^27^ or a decrease of ≥3 Gy in the D98%.^14^, ^19^, ^26^

The 3 Gy threshold for dose changes was adopted from Gan et al.,^19^ who demonstrated that limiting accumulated mean dose rise to within 3 Gy represents a clinically relevant action level for ART in head and neck cancer. The volume reduction thresholds for OARs were informed by Aristophanous et al.^21^ and Zhang et al.,^15^ with moderate adjustments for NPC‐specific anatomy. The target volume reduction thresholds were based on Zhou et al.^27^ and Tang et al.^5^ The PCM thresholds (volume reduction > 5% or dose increase > 1 Gy) were informed by the weekly CT findings of Bhide et al.,^26^ who documented significant volumetric and dosimetric changes in head and neck OARs during radiotherapy. Additional support for the D98% threshold comes from Schwartz et al.^14^

**FIGURE 1:**
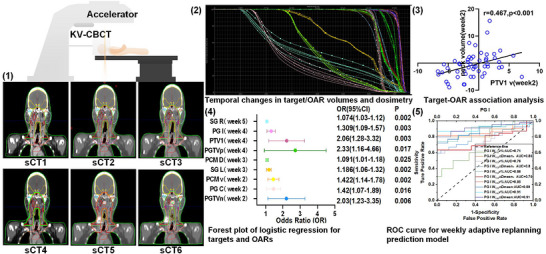
Workflow for deriving an optimal plan adaptation trigger.

This binary endpoint was defined a priori and independently of sCT volumetric analyses. Predictor variables (weekly volume reductions in targets/OARs, dose increases in OARs) were used to model the probability of meeting this pre‐specified replanning condition via binary logistic regression. Model performance was evaluated using ROC analysis. Optimal univariate thresholds for each structure/timepoint were identified by maximizing Youden's Index (J = Sensitivity + Specificity ‐ 1) (Figure 1).

Sensitivity Analysis with Temporally Independent Dosimetric Endpoint: To evaluate the robustness of the predictive framework against a fully independent endpoint, a sensitivity analysis was performed using a temporally separated, purely dosimetric outcome. The binary endpoint was redefined as a ≥3 Gy dosimetric deterioration from baseline, assessed at the treatment week immediately following the predictor measurement (Week N+1). For target volumes (PGTVp), the endpoint was defined as a ≥3 Gy decrease in D98 from baseline; for parotid glands (PG_I, PG_C), the endpoint was a ≥3 Gy increase in mean dose from baseline. Dose‐based predictors were defined as the cumulative dose deviation from baseline measured at Week N. This design ensures complete temporal separation between predictor and outcome, eliminating any potential overlap. The analysis was conducted for PGTVp, PG_I, and PG_C at the monitoring time points identified in the primary findings and at adjacent weeks. PGTVn was excluded because dose deviations alone did not demonstrate significant predictive performance for nodal targets in the original composite endpoint analysis. The established monitoring value of PGTVn resides in its early volumetric response. Receiver operating characteristic curves were generated, and AUC values were compared descriptively with those from the original composite endpoint analysis.

## 3 RESULTS

### 3.1 Patients

From an initial cohort of 55 NPC patients, three were excluded for missing ≥2 weekly CBCT scans during treatment, yielding a final analytical sample of 52 patients.

### 3.2 Volumetric variations

Significant volumetric changes were observed throughout the treatment course. The Friedman test revealed a significant progressive reduction in the volume of PGTVp and PGTVn shrinking by 2.71% (95% CI: −4.65% to −0.67%; *p *< 0.001) and 13.43% (95% CI: −15.85% to −9.52%; *p *< 0.001) over the 6 weeks (Figures S1a and 2a), respectively. Similarly, PTV1 and PTV2 volumes decreased by 3.99% (95% CI: −5.04% to −2.85%; *p *< 0.001) and 1.22% (95% CI: −2.7% to 0.00%; *p *= 0.001) (Figures S1b and 2b).

**FIGURE 2:**
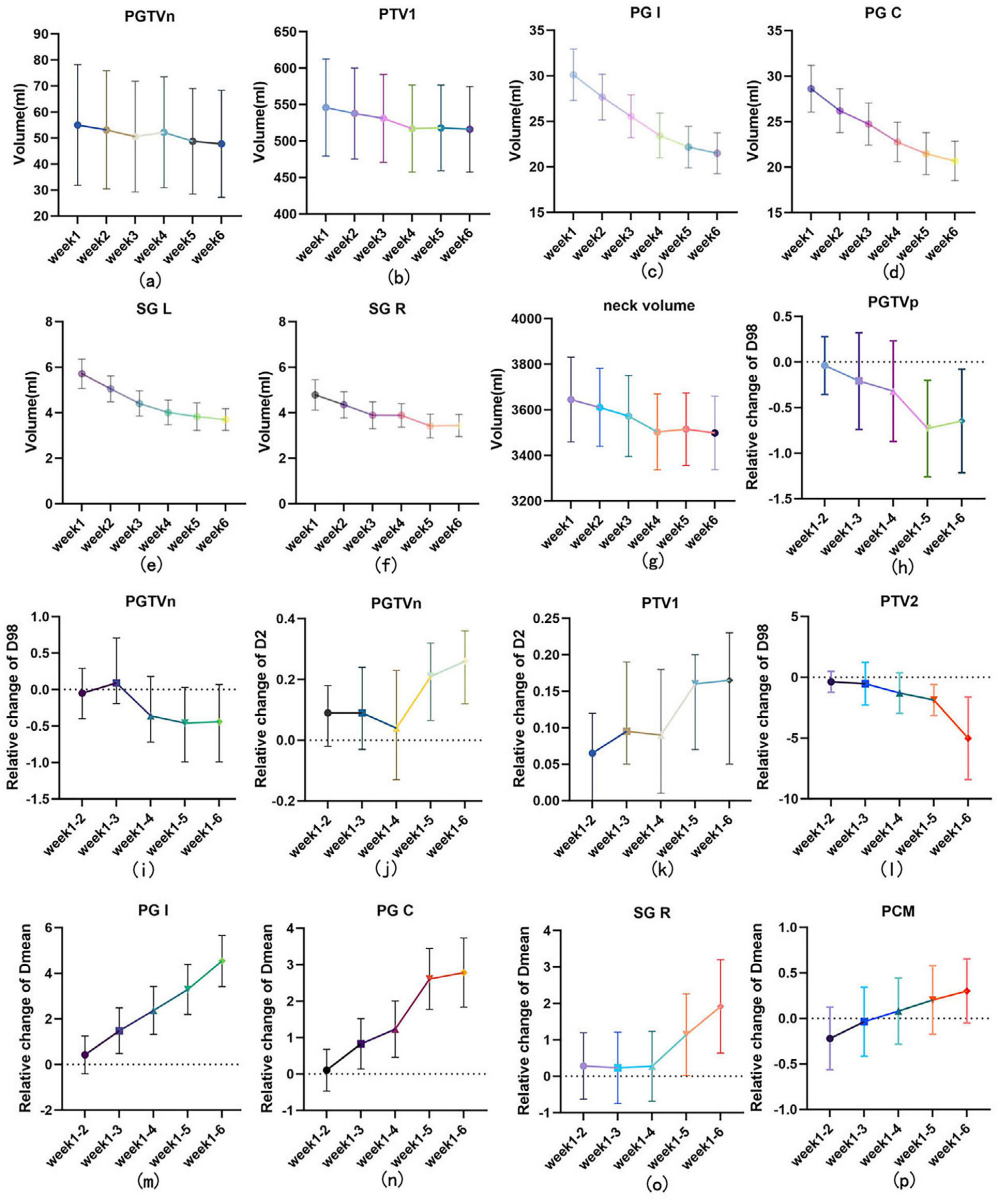
Longitudinal volumetric and dosimetric variations in targets and organs at risk during adaptive radiotherapy for nasopharyngeal carcinoma. (a) Temporal volume changes in nodal gross tumor volume (PGTVn) over 6 weeks of treatment. (b) Volume alterations in planning target volume 1 (PTV1) during radiotherapy. (c) Volume loss in the ipsilateral parotid (PG_I). (d) Volume reduction in the contralateral parotid (PG_C). (e) Lateralized volume changes in the left submandibular gland (SG_L). (f) Volume changes in the right submandibular gland (SG_R). (g) Temporal changes in neck volume during radiotherapy. (h) Cumulative dose reduction in the primary tumor volume (PGTVp) D98 compared to week 1 baseline. (i) Progressive dose reduction in PGTVn D98 relative to week 1 baseline. (j) Progressive increase in PGTVn D2 dose relative to week 1 baseline. (k) Progressive increase in PTV1 D2 dose relative to week 1 baseline. (l) Early and progressive dose reduction in PTV2 D98 relative to week 1 baseline. (m) Progressive dose escalation in PG_I mean dose relative to week 1 baseline. (n) Delayed but significant dose escalation in PG_C mean dose (Dmean) relative to week 1 baseline. (o) Late‐phase dose escalation in SG_R Dmean relative to week 1 baseline. (p) Progressive dose escalation in pharyngeal constrictor muscles (PCM) Dmean relative to week 1 baseline. Data are expressed as percent reduction from baseline (mean ± 95% CI). Statistical comparisons were performed using the Friedman test with post‐hoc analysis (*p* < 0.05 considered significant).

For organs at risk (OARs), bilateral PGs exhibited significant volume loss, with ipsilateral and contralateral reductions of 27.5% (95% CI: −31.03% to −23.05%; *p *< 0.001) and 27.27% (95% CI: −31.49% to −22.66%; *p *< 0.001) by treatment completion (Figure 2c,d). The most rapid decline occurred during weeks 3–4 (ipsilateral: −9.5% [95% CI: −11.88% to −5.62%], *p *= 0.01; contralateral: −8.41% [95% CI: −10.34% to −6.98%], *p *= 0.001). The SGs also atrophied markedly (left: −34.42% [95% CI: −43.83% to −27.2%]; right: −23.89% [95% CI: −41.79% to −17.69%]; both *p *< 0.001) (Figure 2e,f). No significant volume changes were detected in other OARs (brainstem, spinal cord, optic nerves, optic chiasm, eyes, lenses, OC, larynx, or PCM) between weeks 1 and 6 (*p *> 0.05).

### 3.3 Dosimetric variations

9/52 (17%) of patients exhibited a D98% reduction greater than 3 Gy at least once during treatment for PGTVp and PGTVn. Significant dose coverage reductions were observed during the fifth treatment week. For PGTVp, both D98 and V95 decreased markedly (D98: −0.73 Gy [95% CI: −1.26 to −0.2 Gy], *p *= 0.005; V95: −0.20% [95% CI: −0.59% to 0.0%], *p *= 0.002) (Figure 2h and Figure S1c). PGTVn exhibited marginal declines in D98 (−1.03 Gy [95% CI: −2.66 to −0.6 Gy], *p *= 0.057) and V95 (−0.45% [95% CI: −0.86% to −0.04%], *p *= 0.036), accompanied by significant increases in D2 (+0.21 Gy [95% CI: 0.065–0.32 Gy], *p *= 0.001) and heterogeneity index (HI: +1.61% [95% CI: −0.11% to 3.31%], *p *= 0.01) (Figures 2i–j and S1d). PTV1​showed a pronounced rise in D2 (+0.16 Gy [95% CI: 0.07–0.2 Gy], *p *< 0.001) during the same period (Figure 2k). Earlier dosimetric changes were detected in PTV2, with significant reductions in both D98(−0.53 Gy [95% CI: −0.80 to −0.29 Gy]) and V95 (−0.34% [95% CI: −0.56% to −0.15%]) by week 4 (both *p *< 0.001) (Figures 2l and S1e).

At baseline, the Dmean to the PG_I was significantly higher than that to the PG_C (31.13 ± 5.07 Gy vs. 27.85 ± 4.64 Gy; *p *< 0.001). Both glands exhibited progressive dose escalation during treatment (*p *< 0.001), with statistically significant increases emerging at distinct time points: PG I: Dmean increased by 2.37 Gy (95% CI: 1.32–3.42 Gy) by week 4 (*p *< 0.001) (Figure 2m); PG_C: Dmean rose by 2.61 Gy (95% CI: 1.77 to 3.45 Gy) by week 5 (*p *< 0.001) (Figure 2n). Whereas the mean dose (Dmean) to the SG_L remained stable throughout treatment (*p* > 0.05), the SG_R exhibited significant dose escalation, with an increase of 1.92 Gy (95% CI: 0.63–3.2 Gy; *p* = 0.001) by week 6 compared to baseline (Figure 2o). A similar trend was observed in the PCM, where the Dmean showed progressive elevation, rising by 0.29 Gy (95% CI: 0.0 to 0.67 Gy; *p* = 0.001) at week 5 and further increasing to 0.29 Gy (95% CI: 0.18 to 0.65 Gy; *p* < 0.001) by week 6 (Figure 2p).

### 3.4 Correlation analysis

**FIGURE 3:**
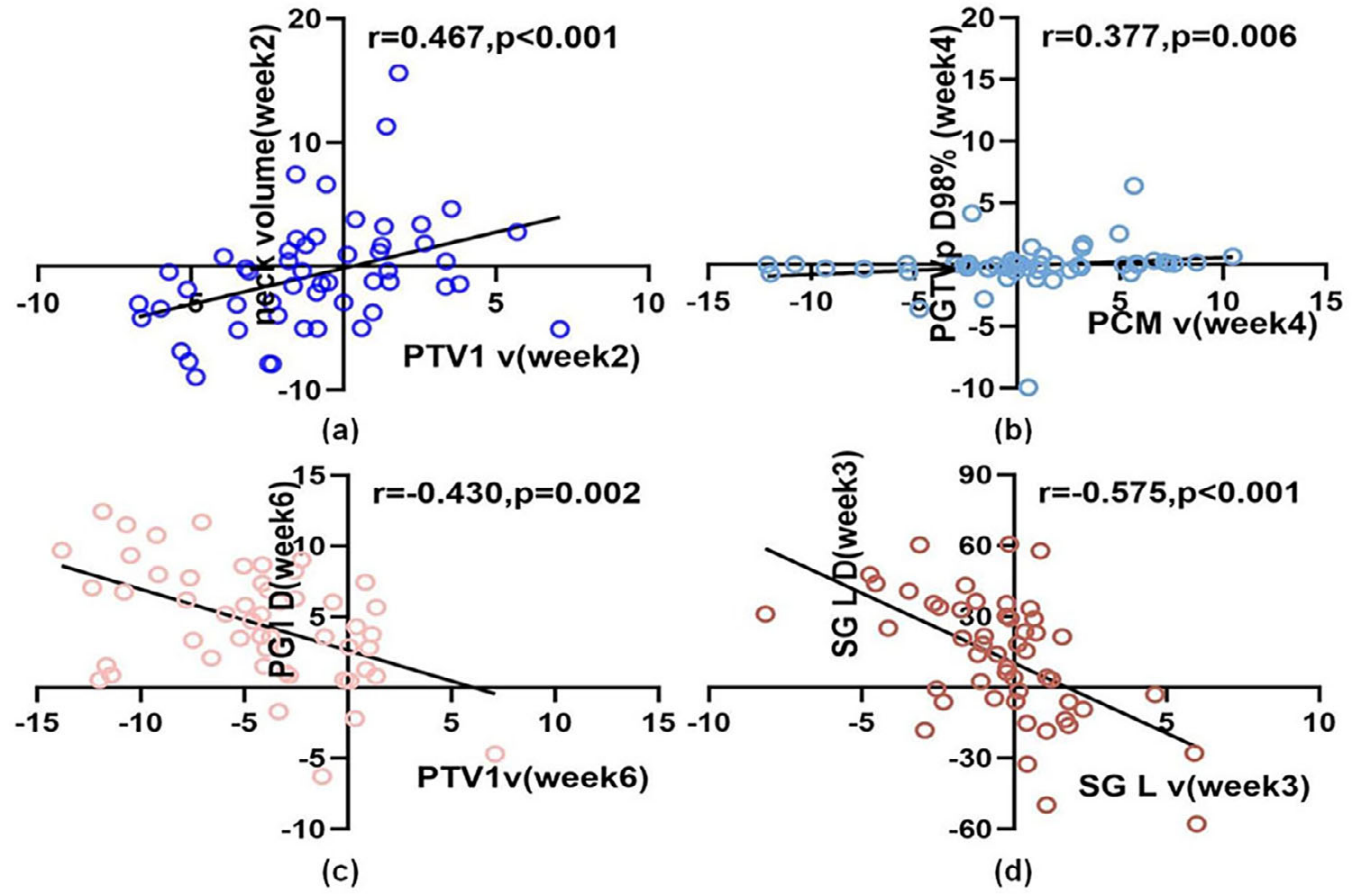
Correlation analysis between volumetric changes and dosimetric parameters for targets and organs at risk. (a) Early correlation between neck volume reduction and planning target volume 1 (PTV1) changes at week 2. (b) Significant dose‐volume correlation between the primary tumor volume (PGTVp) D98% reduction and pharyngeal constrictor muscles (PCM) volume changes during weeks 3–4. (c) Late‐treatment correlation between PTV1 volume reduction and the ipsilateral parotid (PG_I) mean dose escalation during weeks 5–6.​(d) Inverse correlation between the left submandibular gland (SG_L) mean dose increase and volume reduction during weeks 2–3.

Weight loss (measured by neck volume reduction) showed significant correlations with volumetric decreases in both parotid glands. For PG I, correlations were observed during weeks 3–4 (week 3: *r* = 0.352, *p* = 0.11; week 4: *r* = 0.286, *p* = 0.04). PG C also demonstrated significant associations (week 3: *r* = 0.361, *p* = 0.008; week 4: *r* = 0.390, *p* = 0.004). Neck volume reduction exhibited time‐dependent correlations with target volumes: a single association with PGTVn at week 6 (*r* = 0.318, *p* = 0.026), and sustained correlations with PTV1 (peak at week 2: *r* = 0.467, *p* < 0.001(Figure 3a); week 5: *r* = 0.445, *p* = 0.001).

Significant correlations were identified between target volume D98% and salivary gland volumetric changes: PGTVp with PCM at week 4 (*r* = 0.377, *p* = 0.006) Figure 3b); PGTVn with PCM at week 2 (*r* = 0.329, *p* = 0.024), and with PG_C at weeks 3 (*r* = 0.352, *p* = 0.015) and 6 (*r* = 0.336, *p* = 0.021); PTV1 with PCM at week 2 (*r* = 0.286, *p* = 0.040).

The mean dose to the salivary glands showed significant correlations with the volume of the target. PGTVp: week 2, PG C (*r* = ‐0.355, *p* = 0.01). PGTVn: week 5, PCM (*r* = ‐0.336, *p* = 0.021). PTV1: week 5, PG_I (*r* = ‐0.365, *p* = 0.008); week 6, PG_I (*r* = ‐0.430, *p* = 0.002) Figure 3c); week 3, PG_C (*r* = ‐0.316, *p* = 0.022); week 4, PG_C (*r* = ‐0.278, *p* = 0.046); week 5, PG_C (*r* = ‐0.366, *p* = 0.008); week 3, SG_L (*r* = ‐0.349, *p* = 0.011); week 5, SG_L (*r* = ‐0.296, *p* = 0.033); week 2, PCM (*r* = ‐0.343, *p* = 0.013); week 5, PCM (*r* = ‐0.290, *p* = 0.037); week 6, PCM (*r* = ‐0.383, *p* = 0.005).

Time‐dependent correlations were observed between dosimetric changes and volume reductions. Target Volumes: PGTVp: D98% and volume (%) showed a significant correlation at week 3 (*r* = 0.311, *p* = 0.025). PGTVn: significant correlations during weeks 3–4 (week 3: *r* = 0.488, *p* = 0.002; week 4: *r* = 0.308, *p* = 0.035). PTV1: a significant correlation at week 2 (*r* = 0.333, *p* = 0.016). OARs: SG demonstrated significant inverse correlations between Dmean and volume (%): SG L: week 3 (*r* = ‐0.575, *p* < 0.001) (Figure 3d); week 4 (*r* = ‐0.289, *p* = 0.037); week 5 (*r* = ‐0.418, *p* = 0.002). SG R: week 3 (*r* = ‐0.418, *p* = 0.002).

### 3.5 Adaptive timing and trigger thresholds

Binary logistic regression identified a larger volume reduction in the PGTVp and PTV1 observed at the week 4 evaluation was significantly associated with an increased risk of target undosing later in the treatment course (OR = 2.33, 95% CI: 1.16‐4.66, *p* = 0.017; OR = 2.06, 95% CI: 1.28‐3.32, *p* = 0.003). However, an even more pronounced and earlier effect was observed in metastatic lymph nodes. A significant volume reduction in the PGTVn was identified as early as the second week of treatment (OR = 2.03, 95% CI: 1.23‐3.35, *p *= 0.006), suggesting that nodal regions may be more susceptible to rapid anatomical changes and require particularly vigilant monitoring.

**FIGURE 4:**
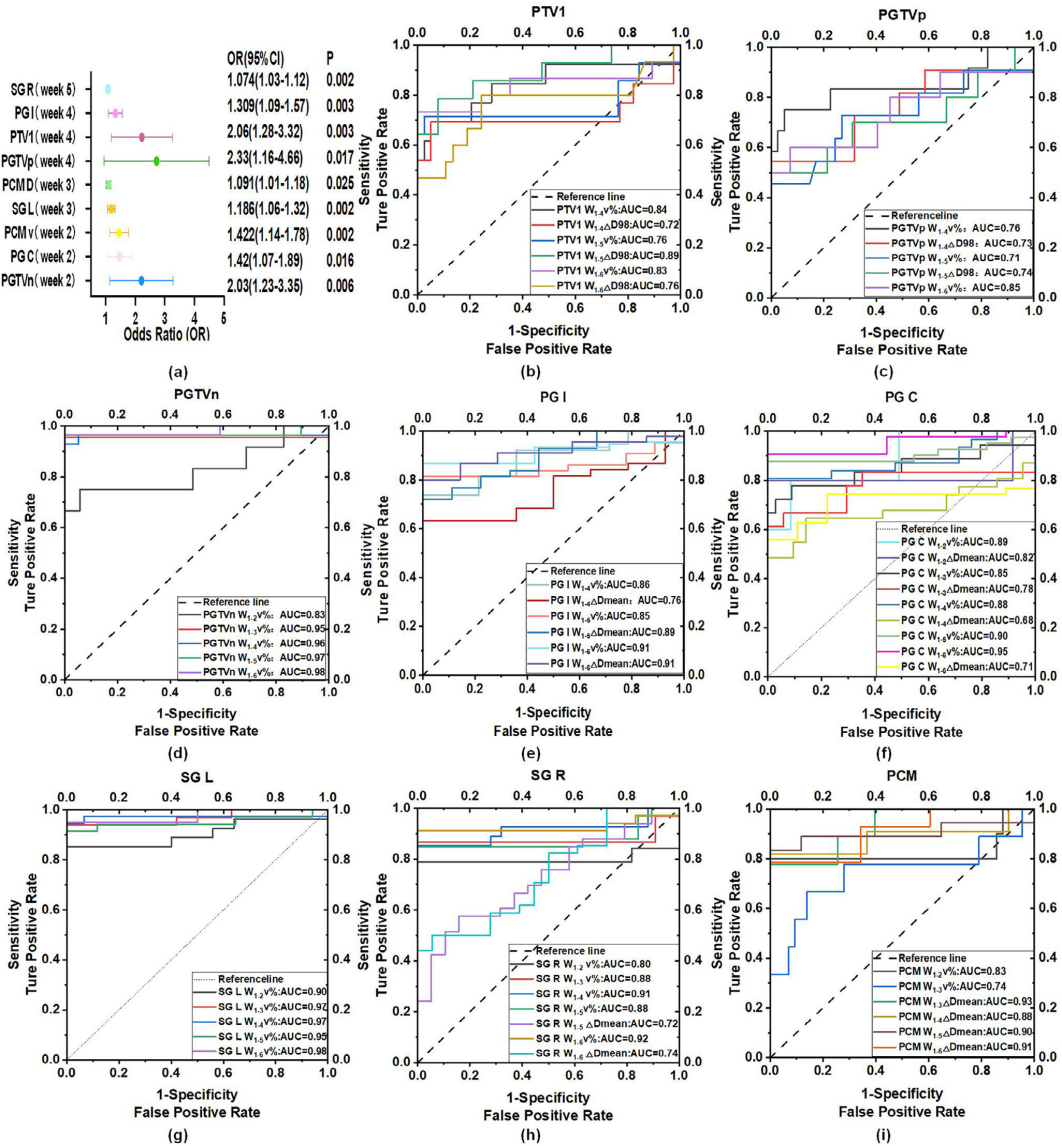
Logistic regression forest plots and receiver operating characteristic curves for adaptive replanning triggers. (a) Forest plot of logistic regression results for targets and organs at risk. (b) receiver operating characteristic (ROC) analysis of planning target volume 1 (PTV1) reduction ≥10% and D98% reduction ≥3 Gy as predictive triggers for adaptive replanning. (c) ROC analysis of the primary tumor volume (PGTVp) reduction ≥10% and D98% reduction ≥3 Gy as predictive triggers for adaptive replanning. (d) ROC analysis of nodal gross tumor volume (PGTVn) reduction ≥10% and D98% reduction ≥3 Gy as predictive triggers for adaptive replanning. (e) ROC analysis of the ipsilateral parotid (PG_I) volume reduction ≥20% and mean dose (Dmean) increase ≥3 Gy as predictive triggers for adaptive replanning. (f) ROC analysis of the contralateral parotid (PG_C) volume reduction ≥20% and Dmean increase ≥3 Gy as predictive triggers for adaptive replanning. (g) ROC analysis of the left submandibular gland (SG_L) volume reduction ≥20% and Dmean increase ≥3 Gy as predictive triggers for adaptive replanning. (h) ROC analysis of the right submandibular gland (SG_R) volume reduction ≥20% and Dmean increase ≥3 Gy as predictive triggers for adaptive replanning. (i) ROC analysis of PCM dose increase ≥1 Gy, or volume reduction > 5% as predictive triggers for adaptive replanning.

A time‐dependent pattern of volumetric changes was observed across various organs at risk (OARs). Notably, the PG exhibited an early but lateralized response. A significant volume reduction in the contralateral gland was observed as early as the second week (OR = 1.42, 95% CI: 1.07‐1.89, *p *= 0.016). In contrast, a significant reduction in the ipsilateral gland was not detected until the fourth week (OR = 1.309, 95% CI: 1.09‐1.57, *p *= 0.003). The SGs showed a more delayed and lateralized response; the left gland's volume reduction became significant in the third week (OR = 1.186, 95% CI: 1.06‐1.32, *p *= 0.002), while the right gland's change was not significant until the fifth week (OR = 1.074, 95% CI: 1.03‐1.12, *p *= 0.002).​For the PCM, a sequential change was identified: significant volume reduction occurred early, by the second week (OR = 1.422, 95% CI: 1.14‐1.78, *p *= 0.002), which was subsequently followed by a significant increase in mean dose during the third week (OR = 1.091, 95% CI: 1.01‐1.18, *p *= 0.025) (Figure 4a).

ROC analysis for week 4 PGTVp reduction yielded an AUC of 0.76 (Figure 4c). The optimal trigger threshold was a 10% volume reduction at week 4, which predicted the need for adaptation with a sensitivity of 54% and specificity of 98% (Youden's index = 0.52). The PTV1 was best predicted by a smaller reduction of ≥7% at the same time point (week 4), which achieved a higher AUC of 0.84, with 69% sensitivity and 95% specificity (Youden's index = 0.64) (Figure 4b). In contrast, the most predictive changes for PGTVn occurred much earlier. A volume reduction of ≥8% at week 2 was the optimal trigger, demonstrating high predictive accuracy (AUC = 0.83, sensitivity = 75%, specificity = 94%, Youden's index = 0.69) (Figure 4d).

The timing and accuracy of predictive models for OAR changes were highly structure‐specific. The most predictive model was for the left submandibular gland (Week 3, ≥19% reduction, AUC = 0.97) (Figure 4g), demonstrating near‐perfect discrimination. An earlier, highly accurate signal was derived from the contralateral parotid (Week 2, ≥15% reduction, AUC = 0.89) (Figure 4f). A clear lateralized and delayed pattern was observed for the SGs, with the right gland's optimal threshold occurring at week 5 (≥18% reduction, AUC = 0.88) (Figure 4h). For the pharyngeal constrictors, a dose‐volume sequential model was established, where an early volume loss (Week 2, ≥4%) powerfully predicted a subsequent dose increase (Week 3, ≥0.9 Gy, AUC = 0.93) (Figure 4i).

### 3.6 Sensitivity analysis with independent dosimetric endpoint

The sensitivity analysis using the temporally independent, purely dosimetric endpoint confirmed the predictive patterns identified in the primary analysis (Table S1). The dose‐based predictors maintained discriminative capacity across all evaluated structures, with AUC values ranging from 0.61 to 0.95. A consistent temporal gradient was observed: for PG_C, the AUC improved from 0.66 (Week 2 to Week 3) to 0.79 (Week 3 to Week 4) and 0.87 (Week 4 to Week 5) (Figure S2a); for PGTVp, from 0.61 (Week 4 to Week 5) to 0.91 (Week 5 to Week 6) (Figure S2b); and for PG_I, from 0.84 (Week 4 to Week 5) to 0.95 (Week 5 to Week 6) (Figure S2c). The monitoring weeks identified in the primary analysis yielded measurable AUCs in the sensitivity analysis, with AUC values increasing at later weeks. Parotid glands consistently showed higher AUC values than PGTVp. Because the original and sensitivity analyses used different endpoint definitions and timeframes, AUC values were not directly compared across analyses.

## 4 DISCUSSION

In this study, we report an optimized offline ART workflow that employs DL‐generated sCTs for plan evaluation on a conventional linac, aiming to improve target coverage and OAR sparing in NPC. The quantitative results from this workflow defined potential triggers and optimal timing for plan adaptation, offering insights into addressing the critical clinical challenge of determining when to intervene to balance treatment efficacy and clinical workload.

The heterogeneous temporal patterns observed in tumor and organ volumes are driven by significant biological and physical mechanisms. From a biological standpoint, the more rapid shrinkage of PGTVn compared to PGTVp can be attributed to the tumor microenvironment.^28^, ^29^, ^30^ PGTVn are often characterized by lymphocyte‐rich, hypervascularized environments,^31^ which could potential enhance radiosensitivity and lead to rapid volumetric regression. This observation aligns with documented spatial heterogeneous radiosensitivity within head and neck tumors, where regional variations in the microenvironment influence treatment response.^32^ Concurrently, physical and dosimetric consequences arise from these anatomical changes. For salivary glands, volumetric reduction is strongly correlated with the radiation dose delivered.^33^ Specifically, ipsilateral glands showing more pronounced volume loss are associated with patient‐reported xerostomia, highlighting the clinical relevance of dose‐volume relationships.^34^, ^35^ This phenomenon is further explained by an earlier study,^26^ which used weekly CT scans to identify a significant 2.7 Gy increase in mean dose to the ipsilateral gland by week 4. This dose escalation was attributed to cumulative CTV shrinkage and the subsequent medial displacement of the gland into the high‐dose region. A similar dose‐volume relationship was observed in the PCM, where progressive atrophy erodes the anatomical buffer zone separating it from the high‐dose PTV, leading to dose escalation. These examples show that volumetric metrics alone cannot capture spatially resolved dosimetric risks, highlighting the need for complementary dosimetric validation to assess the impact of anatomical changes during radiotherapy.

The trigger thresholds proposed in this study, such as an ≥8% volume reduction for PGTVn or ≥15% for the contralateral parotid, align with the evolving literature on adaptation triggers. Previous investigations have suggested various action levels, including a parotid volume decrease > 7%^21^ or a universal 5% threshold.^22^ However, our study advances this concept by employing ROC methodology to correlate volumetric changes with final dosimetric outcomes. This analytical approach enabled the development of a set of structure‐specific thresholds, which demonstrated strong predictive performance (e.g., an AUC of 0.83 for PGTVn). Consequently, this provides a more refined and evidence‐based strategy for identifying patients who may benefit from ART.

Our findings on the dosimetric impact of target volume reduction align with the existing literature,^26^ which recommends replanning by week 2 to mitigate the significant decrease in minimum doses to PTV_1_ and PTV_2_. A key concern underlying this recommendation is whether such volume reduction compromises tumor control. This concern was directly addressed by the work of Zhou et al.,^27^ who demonstrated that appropriately shrinking the target volume during ART did not increase recurrence risk within the “reduced regions.” Their finding that the vast majority of recurrences remain classical “in‐field” failures provides strong evidence supporting the safety of reduced‐volume radiotherapy. The safety of this approach is further supported by recent clinical evidence. For instance, the phase III trial by Sun et al.^36^ established the role of induction chemotherapy for locoregionally advanced NPC, while Tang et al.^5^ confirmed that reduced‐volume radiotherapy based on post‐induction tumor volume was non‐inferior to conventional radiotherapy in locoregional control, while significantly reducing toxicity and improving quality of life. In our study, we observed a significant correlation between target volume reduction and a decrease in the minimum dose (D98%) to the PTV. This finding provided a potential dosimetric explanation for the “in‐field recurrences” reported by Zhou et al.,^27^ suggesting that “cold spots” arising from target volume reduction could compromise tumor control probability. Based on this collective evidence, we propose that a target volume reduction of ≥10% serves as a trigger for replanning. This strategy is fundamentally a proactive adaptive strategy designed to ensure the tumor target consistently receives an adequate radiation dose. By promptly identifying and correcting potential cold spots, this approach aims to safeguard tumor control probability as effectively as possible.

Our proposed triggers are strongly supported by the large‐scale simulation study from Gan et al.,^19^ which provides crucial external validation on the optimal timing for adaptation. In their comprehensive analysis of 110 H&N patients, they identified the third week as the most frequent single timepoint for replanning. More importantly, their analysis demonstrated that two replannings were sufficient to limit dosimetric drift, with schedules such as weeks 2 & 4 or 3 & 5 keeping the accumulated mean dose increase below 3 Gy for 99% of patients across 19 OARs. This finding directly corroborates the timing of our own strategy. Our protocol, which triggers replanning for PGTVn at week 2 and PGTVp at week 4, effectively bookends this critical adaptation window. Furthermore, our inclusion of early OAR triggers (e.g., contralateral parotid at week 2) aligns with this paradigm of proactive intervention to control dosimetric changes.

A key strength of our study lies in an integrated, automated workflow, “sCT + auto‐segmentation + GPU Monte Carlo dose recalculation”, that directly addresses a major limitation of conventional CBCT of its inability to be used for accurate dose calculation. While comprehensive oART solutions like MR‐Linacs or Ethos integrate imaging, re‐optimization, and treatment delivery, they require dedicated, expensive platforms and significant time investment (median 46 minutes for MR‐Linac^23^ and 20–30 minutes for Ethos^12^). In contrast, our proposed workflow focuses specifically on rapid dosimetric evaluation. As a complementary approach, it dramatically lowers the barrier to assessing plan quality without the need for full re‐optimization, completing the evaluation in under 3 minutes. This efficiency, shared by similar tools like ArcherQA, makes advanced ART feasible for the vast global network of conventional C‐arm linacs, thereby promoting its routine clinical adoption.

Our study has several limitations that must be considered. First, it is based on a retrospective simulation of treatment plan execution rather than a prospective clinical evaluation, which precludes a direct assessment of clinical benefits. Second, the single‐center nature of our data inherently limits the generalizability of our findings. Third, the potential for circularity in endpoint definition warrants caution. Since the predictor variables (volumetric changes) and the endpoint criteria (replanning required based on dosimetric shifts) involve the same anatomical structures, the reported ROC thresholds should be interpreted as context‐dependent monitoring parameters rather than generalizable clinical triggers. A sensitivity analysis using a temporally independent, purely dosimetric endpoint confirmed that dose‐based predictive patterns were preserved across all evaluated structures (AUC range: 0.61–0.95), with a consistent temporal gradient toward higher predictability at later treatment weeks. Nevertheless, this study demonstrates the feasibility of using an automated sCT pipeline to track dosimetric trajectories associated with replanning needs. Future prospective validation using independent endpoints (e.g., physician consensus) is required to establish these thresholds as formal clinical decision rules. Finally, the accuracy of our quantitative assessments, particularly for dose and volume parameters, is critically dependent on the quality of the sCT data, which may introduce potential biases.

To address these limitations and advance the field, our future work will focus on several key areas. We will prioritize prospective, multi‐center validation of our proposed triggers to confirm their robustness and clinical utility. This will be coupled with the integration of Normal Tissue Complication Probability models to enable truly toxicity‐driven adaptation. Furthermore, we aim to develop AI‐based models for predicting patient‐specific anatomical changes, and to correlate these predictive triggers with actual clinical outcomes. These efforts are essential for realizing a truly personalized and predictive ART workflow.

## 5 CONCLUSION

This study established a structured, evidence‐based framework for ART in NPC, leveraging weekly sCT to map the distinct temporal patterns of anatomical and dosimetric change. Our analysis revealed critical, structure‐specific triggers that dictate optimal intervention windows: an early adaptation at week 2 for nodal targets and the contralateral parotid, and a later one at week 4 for primary targets and the ipsilateral parotid. Beyond these key timepoints, we developed a dose–volume sequential model for pharyngeal constrictors and identified highly predictive thresholds for submandibular glands, paving the way for truly proactive replanning. The resulting protocol offers a practical roadmap for implementing precision ART monitoring on conventional C‐arm linacs, with the identified temporal patterns and thresholds serving as candidate predictors pending prospective validation.

## AUTHOR CONTRIBUTIONS

Zhenmei Cao designed the whole experiment and wrote the manuscript. Chun Wu collected and analyzed the data. Lixiang Han, Tingting Duan, Fengyu Zhang, Lansheng Zhang, and Bin Wang designed the plans in the study. Weijie Lei and Xinye Ni supervised the whole study. All co‐authors have reviewed and approved the final manuscript.

## CONFLICT OF INTEREST STATEMENT

The authors declare no conflicts of interest.

## Supporting information



Supporting Information

Supporting Information

## Data Availability

The data that support the findings of this study are available from the corresponding author upon reasonable request.
