# Supplementary material for: Synthetic CT‐enabled weekly adaptive radiotherapy for nasopharyngeal carcinoma: Optimizing plan adaptation triggers through volumetric–dosimetric monitoring
Source: J Appl Clin Med Phys. 2026 Jul 1;27(7):e70676. doi: 10.1002/acm2.70676 (PMC13322649; doi:10.1002/acm2.70676)
Supplement: Supplementary file 1 — Supporting Information [file ACM2-27-e70676-s002.docx]

Table S1. Comparison of predictive performance between the original analysis (same-week, composite endpoint) and the sensitivity analysis (next-week, purely dosimetric endpoint).

| Structure | Predictor Week | Original Analysis (Same-Week) |  | Sensitivity Analysis (Next-Week) |  |
| --- | --- | --- | --- | --- | --- |
|  |  | Predictor → Endpoint | AUC | Predictor → Endpoint | AUC |
| **PGTVp** | Week4 | W1-4 dose → W1-4 composite | 0.74 | W1-4 dose → W1-5 ≥3 Gy | 0.61 |
| PGTVp | Week5 | W1-5 dose → W1-5 composite | 0.74 | W1-5 dose → W1-6 ≥3 Gy | 0.91 |
| PG I | Week4 | W1-4 dose → W1-4 composite | 0.76 | W1-4 dose → W1-5 ≥3 Gy | 0.84 |
| PG I | Week5 | W1-5 dose → W1-5 composite | 0.89 | W1-5 dose → W1-6 ≥3 Gy | 0.95 |
| PG C | Week2 | W1-2 dose → W1-2 composite | 0.82 | W1-2 dose → W1-3 ≥3 Gy | 0.66 |
| PG C | Week3 | W1-3 dose → W1-3 composite | 0.78 | W1-3 dose → W1-4 ≥3 Gy | 0.79 |
| PG C | Week4 | W1-4 dose → W1-4 composite | 0.68 | W1-4 dose → W1-5 ≥3 Gy | 0.87 |

*Note:* The original analysis used a composite (volume + dose) endpoint assessed within the same treatment week. The sensitivity analysis used a purely dosimetric endpoint (≥3 Gy deviation from baseline) assessed at Week N+1, with dose-based predictors measured at Week N. This design ensures complete temporal separation between predictor and outcome. PGTVn was excluded because dose deviations did not achieve significant predictive performance for nodal targets in the original composite endpoint analysis. As the two analyses differ in endpoint definition and temporal framework, AUC values are not directly comparable. The sensitivity analysis serves as an independent conceptual replication.
