# Supplementary material for: Synthetic CT‐enabled weekly adaptive radiotherapy for nasopharyngeal carcinoma: Optimizing plan adaptation triggers through volumetric–dosimetric monitoring
Source: J Appl Clin Med Phys. 2026 Jul 1;27(7):e70676. doi: 10.1002/acm2.70676 (PMC13322649; doi:10.1002/acm2.70676)
Supplement: Supplementary file 2 — Supporting Information [file ACM2-27-e70676-s001.docx]

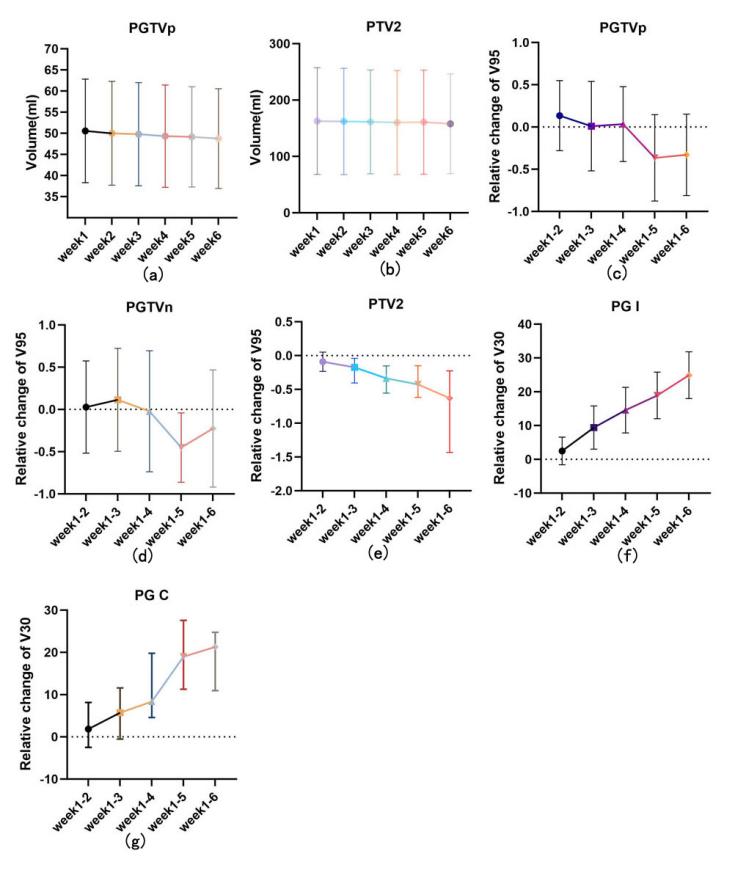


Figure S1. Longitudinal volumetric and dosimetric variations in targets and organs at risk during adaptive radiotherapy for nasopharyngeal carcinoma.

(a) Temporal volume changes in primary tumor volume (PGTVp) over 6 weeks of treatment. (b) Volume alterations in planning target volume 2 (PTV2) during radiotherapy. (c) Temporal changes in PGTVp V95 coverage relative to week 1 baseline. (d) Temporal changes in PGTVn V95 volume coverage relative to week 1 baseline. (e) Early and progressive change in PTV2 V95 coverage relative to week 1 baseline. (f) Progressive change in PG_I V30 dose volume relative to week 1 baseline. (g) Delayed but significant V30 dose-volume escalation in PG_C relative to week 1 baseline. Expressed as percent reduction from baseline (mean ± 95% CI), Statistical comparisons were performed using Friedman test with post-hoc analysis (p < 0.05 considered significant), Positive values indicate an increase, negative values indicate a decrease from the week 1 baseline.

**
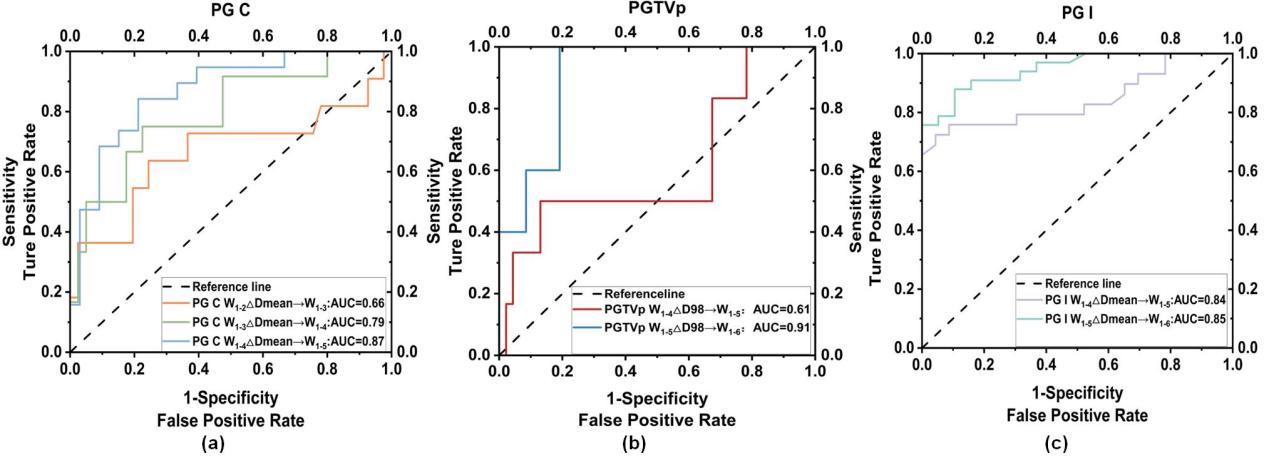
**

Figure S2. Receiver operating characteristic (ROC) curves for the sensitivity analysis using a temporally independent, purely dosimetric endpoint.

(a) ROC analysis of PG_C cumulative dose deviation (W1-2, W1-3, and W1-4) as predictors of a ≥3 Gy mean dose increase in the subsequent week (W1-3, W1-4, and W1-5, respectively; AUC = 0.66, 0.79, and 0.87). (b) ROC analysis of PGTVp cumulative D98 deviation (W1-4 and W1-5) as predictors of a ≥3 Gy D98 decrease in the subsequent week (W1-5 and W1-6, respectively; AUC = 0.61 and 0.91). (c) ROC analysis of PG_I cumulative dose deviation (W1-4 and W1-5) as predictors of a ≥3 Gy mean dose increase in the subsequent week (W1-5 and W1-6, respectively; AUC = 0.84 and 0.95).
